# Revisiting the Advances in Isolation, Characterization and Secretome of Adipose-Derived Stromal/Stem Cells

**DOI:** 10.3390/ijms19082200

**Published:** 2018-07-27

**Authors:** Navneet Kumar Dubey, Viraj Krishna Mishra, Rajni Dubey, Yue-Hua Deng, Feng-Chou Tsai, Win-Ping Deng

**Affiliations:** 1 Ceramics and Biomaterials Research Group, Advanced Institute of Materials Science, Ton Duc Thang University, Ho Chi Minh City 700000, Vietnam; navneet.kumar.dubey@tdt.edu.vn; 2 Faculty of Applied Sciences, Ton Duc Thang University, Ho Chi Minh City 700000, Vietnam; 3 Applied Biotech Engineering Centre (ABEC), Department of Biotechnology, Ambala College of Engineering and Applied Research, Ambala 133101, India; viraj.krishna@gmail.com; 4 Graduate Institute Food Science and Technology, National Taiwan University, Taipei 10617, Taiwan; rajni.nitrkl@gmail.com; 5 Stem Cell Research Center, Taipei Medical University, Taipei 11031, Taiwan; yuehuahua828@gmail.com; 6 Department of Life Science, Fu Jen Catholic University, New Taipei City 24205, Taiwan; 7 School of Dentistry, College of Oral Medicine, Taipei Medical University, Taipei 11031, Taiwan; 8 Department of Basic medicine, Fu-Jen Catholic University, New Taipei City 24205, Taiwan

**Keywords:** adipose tissue, adipose-derived stem cells, secretome, regenerative therapy

## Abstract

Adipose-derived stromal/stem cells (ASCs) seems to be a promising regenerative therapeutic agent due to the minimally invasive approach of their harvest and multi-lineage differentiation potential. The harvested adipose tissues are further digested to extract stromal vascular fraction (SVF), which is cultured, and the anchorage-dependent cells are isolated in order to characterize their stemness, surface markers, and multi-differentiation potential. The differentiation potential of ASCs is directed through manipulating culture medium composition with an introduction of growth factors to obtain the desired cell type. ASCs have been widely studied for its regenerative therapeutic solution to neurologic, skin, wound, muscle, bone, and other disorders. These therapeutic outcomes of ASCs are achieved possibly via autocrine and paracrine effects of their secretome comprising of cytokines, extracellular proteins and RNAs. Therefore, secretome-derivatives might offer huge advantages over cells through their synthesis and storage for long-term use. When considering the therapeutic significance and future prospects of ASCs, this review summarizes the recent developments made in harvesting, isolation, and characterization. Furthermore, this article also provides a deeper insight into secretome of ASCs mediating regenerative efficacy.

## 1. Introduction

The self-renewal and differentiation potential of adipose-derived stromal/stem cells (ASCs) have accelerated the progress in regenerative therapy. In the previous literatures, a variety of terms have been used for these cells, such as adipose-derived adult stromal cells, adipose-derived adult stem (ADAS) cells, adipose-derived stromal cells (ADSC), adipose stromal cells (ASC), adipose mesenchymal stem cells (AdMSC), preadipocytes, processed lipoaspirate (PLA) cells, and adipose-derived stromal/stem cells (ASCs); however, to address this discrepancy, the International Fat Applied Technology Society (IFATS) reached a consensus to refer them as adipose-derived stromal/stem cells (ASC) [1]. These cells are mainly present in perivascular region of all tissue and organs, including white adipose tissues [2,3,4,5]. The higher abundance of ASCs in these areas from which they could be easily harvested via minimally invasive procedures make them a suitable agent in cell-based therapy [5]. Aesthetic and economical liposuction surgeries are less painful and provide rich source of ASCs and progenitor cells in large quantity as compared to harvesting bone marrow stem cells (BMSCs) [6,7]. ASCs have been reported for its pluripotency/plasticity into various cells, such as chondrocytes, osteoblasts, myocytes, adipocytes, neural cells, and epithelial cells [8,9,10]. Therefore, their regenerative potential have been explored in the treatment of various diseases, such as diabetes and related complications, osteoarthritis, cardiovascular diseases [11,12,13], nerve regeneration and neurological disorders [10,14,15], skin aging [16], ischemic limb disease [17], skin burn, and wound healing [18,19,20]. Along with differentiation potential of ASCs, the exhibited paracrine activity, and secretion of growth and signaling factors enhance their clinical significance [21]. It is reported that ASCs maintain their phenotypic characteristics, differentiation potential, and proliferation capacity even after 25 passages [22]. This indicates their reduced frequency of passaging and hence the low risk of cellular senescence [23,24]. In the recent years, the intense research has focused on isolation and characterization of ASCs from various adipose tissue sources of animal models and human. These ASCs are present in stromal vascular fraction (SVF) along with other cells, such as endothelial, hematopoietic, and other cells [23,25]. After lipoaspiration, the adipose tissue is digested with collagenase and subcultured to obtain the sufficient number of cell populations [26]. Thereafter, the cell proliferation/viability is determined and the cells are further assessed for the presence of mesenchymal stem cell characteristics, such as cell surface markers in form of cluster of differentiation (CD) [5,6,7,25,27,28], and their multi-lineage differentiation potential, which is determined by culturing them in specific induction media.

Contemplating the importance of regenerative potential of ASCs; this review article comprehensively summarizes the isolation, characterization, and differentiation methodologies of ASCs from various sources for their possible use in regenerative therapy.

## 2. Adipose Tissues as Source of ASCs

Adipose tissues are a rich and popular source of adult stem cells [29,30]. They are also involved in homeostasis, metabolism regulation, and aging processes [31]. These tissues are derived from mesenchyme and mainly constitute stem cells, endothelial cells, collagen, resident monocytes/macrophages, lymphocytes, fibroblasts, vascular smooth muscle cells, preadipocytes, and adipocytes [26,32,33,34,35]. Adipose tissues are classified into three groups, namely, white adipose tissue (WAT), bone marrow adipose tissue (BMAT), and brown adipose tissue (BAT) in mammals [36,37,38,39]. Both, the BAT and WAT contain lipolytic and lipogenic functions and are involved in energy accumulation and dissipation, respectively [36]. However, BATs are larger in size and rich in mitochondria than WAT and possess a specific uncoupling protein-1 (UCP-1), a mitochondrial ion carrier [40]. The existing color of BAT is due to the high number of vascularization, cytochromes, and mitochondria, which is responsible for high energy dissipation along with other dedicated proteins, such as UCP-1 [41,42]. On the other hand, WAT is found as subcutaneous and visceral depots that are not involved in metabolic disorder due to high number of young adipocytes and adipose turnover [43,44]. The role of WAT in metabolism regulation is critical and has been shown that several metabolic disorders such as hyperglycemia, diabetes, hypertension, liver disease, hyperlipidemia, etc. are generally caused by an imbalance in adipocytes [45]. In addition, BAT is considered as source of heat as it catalyzes energy uncoupling, dissipation, and mitochondrial biogenesis [46]. Heterogeneity and plasticity are the characteristics of adipose tissues that depend on species and source of fat depots [42]. Adipose tissues are appealing due to their higher abundance in stem cells and ease of harvest when compared to bone marrow [42]. However, the ASCs yield is influenced by various factors, such as age, location of adipose tissue, harvesting techniques, and species [47,48,49]. BAT is mainly found in axillary, perirenal, periadrenal regions, and cervical in fetus and neonate; however, this tissue transformed mostly into WAT in adults [50,51]. Whereas, the human WAT is distributed at various sites, such as subcutaneous region of abdomen, thighs and buttocks, intestines, perirenal, omentum, retro-orbital space, and bone marrow [52,53]. Subcutaneous depots are localized at superficial and deep region of abdomen and are considered superior source of stem cells when compared to other fat depots [52]; however, superficial abdominal adipose tissues has showed an enhanced multipotency and stemness characteristics [54]. Standard en bloc resection and lipospiration are the two most common surgical procedures that are used to harvest adipose tissues [52]. Further, though the efficacy of adipose tissues site on ASC yield and its characteristics is not explicitly established, a few seminal studies demonstrated that abdominal adipose tissue is rich in ASCs as compared to other sites, such as hip, thigh, femoral, axilla, and flank regions [25,47,55]. Furthermore, another comparative study implied that superficial adipose tissue is a better source of stromal vascular fraction (SVF) [56]. These bodies of evidence indicate that superficial abdominal adipose tissue is a prospective source of ASCs. However, in contrast to the above studies, a recent report revealed a significantly higher yield of ASCs and SVF from adipose tissues of inner as well as outer thigh when compared to those of abdominal, waist, and inner knee regions [57]. In another study, Khojasteh et al. suggested that, when compared to abdomen and hip regions of both male and female donors, the buccal fat pad seems more promising source of ASCs for regenerating bone tissues [58]. On the other hand, no significant differences on yield characteristics and viability have also been observed among ASCs of abdomen, thigh, or hip [59]. Along with the donor site, the factors, such as gender and age, have also been extensively evaluated and need to be considered during harvesting of adipose tissues to isolate ASCs [60]. In a rabbit model, the aging induced suppression of ASCs yield and adipogenic potential was evident with no significant effect on their osteogenic and clonogenicity [61]. A recent systemic review has reported an inihibited proliferation and differentiation potential of ASCs with advancing age [62]; however, this phenomenon was not extensively uniform throughout. A comparative study concluded that yield and characteristics of human orbital adipose derived stem cells remain constant among young and aged donor [63]. Similarly, no significant effect of aging on ASC yield and therapeutic potential of ASCs was observed from adult to elderly stem cells [64]. This might be attributed to no influence of aging on the cellular senescence and ASC yield from subcutaneous adipose tissue, thereby gaining promising potential for regenerative therapy [65]. On contrary, Lee et al. found the higher cell population doubling levels and differentiation potential of ASCs among younger dog when compared to older ones, indicating that the age of donor is an important factor in cell-based therapy [66].

Coleman’s technique, direct excision, and liposuction are the common harvesting techniques that are used in collection of adipose tissues [49]. The collection site and procedure followed in above techniques affects the yield and characteristics of ASCs [67]. However, no significant effect on ASCs yield and differentiation potential through direct resection and liposuction had also been reported. Further, the ASCs obtained through ultrasound-assisted liposuction are lower in yield and proliferative potential compared to resection and tumescent liposuction methods [59]. Notwithstanding, the liposuction seems better harvesting technique, and yield more homogenous ASCs than the resection technique [68]. However, the pattern of expressed genes from ASCs isolated by liposuction indicate their enhanced endodermal differentiation; whereas, ASCs isolated by resection had tendency of mesoderm and ectoderm differentiation [68]. Taken together, though the recent studies have established several factors that might affect ASCs yield, viability, and characteristics, an intense investigation is needed to gain a deeper insight on the role of factors on quality and quantity of ASCs.

### 2.1. Harvesting of Adipose Tissues

Harvesting adipose tissue is the first step in isolation of ASCs. The three general techniques liposuction, resection, and Coleman are used to harvest adipose tissues from human fat sites; of which, the liposuction results in better cellular yield and viability than others [69]. Liposuction is one of the most common and increasingly used surgical operation being carried out by plastic and reconstructive surgeons since several past years to restructure body contour to improve aesthetic looks and in treatment of pathologies in reconstructive surgery [70]. Further, though the various sites are targeted to harvest adipose tissues, subcutaneous regions are considered as the most appropriate choice [70,71]. Liposuction is generally carried out Recently, Arpad and Giorgio Fischer has developed a novel method of liposuction in which a blunt hollow cannula is attached to a suction source to extract adipose tissues with lesser complication. This dry liposuction technique was further modified as wet/tumescent liposuction to decrease the effect of hemorrhagic risk and the associated complexity through bringing down the bleeding level <1% compared to 30% of dry liposuction [72]. In the wet/tumescent technique, firstly the Klein solution (0.05% lidocaine, 1:1,00,000 epinephrine, and 10 mL sodium bicarbonate per 1000 mL saline) or saline solution containing local anesthetic agent and/or epinephrine is injected at the target site to reduces blood loss and enhance the safety of the procedures [73]. Thereafter, the adipose tissues are harvested by using cannula and syringe of different sizes.

Besides, vaccum or syringe aspirations are the most commonly followed techniques during fat harvesting procedures [74,75]. The increased vacuum increases the aspiration rate; however, a very high pressure may disrupt structural integrity of ASCs and other cells [76,77]. Additionally, cannula size and types of syringe needles also might affect the cell yield, size, and viability of harvested fat [78,79,80]. However, a study by Campbell et al. reported that if the needle size is greater than 20 gauge, it exerts no significant effect on adipocyte morphology and metabolism [81]. In contrast to above studies, no significant effect of multi-perforated cannula with the Coleman 3 mm aspiration cannula was observed on cell viability or size of fat tissues [82,83]. Ultrasound and laser-assisted liposuction are the other two approaches to harvest fat with enhanced accuracy and safety during procedures [84,85,86]. Besides, the Coleman developed fat harvesting techniques using syringe, cannula, and centrifuge in which an incision is made at the target site and injected with 1 cc solution per cm^3^ of fat to be harvested [87].

### 2.2. Isolation of ASCs from Harvested Adipose Tissues

**Figure 1:**
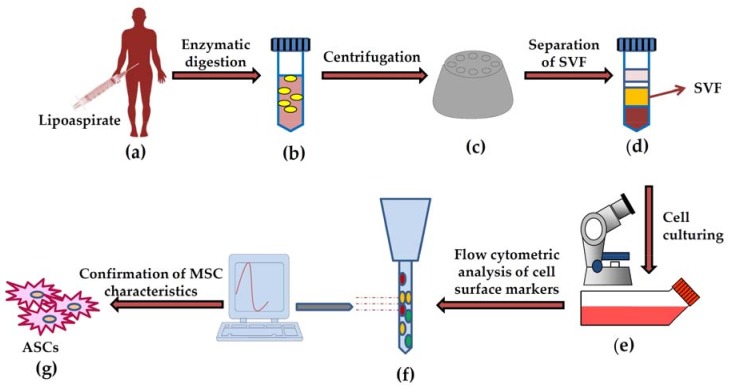
Schematic representation of process for harvesting, isolation and characterization of adipose derived stem cells (ASCs). Adipose tissues are harvested through liposuction, enzymatically digested; and centrifuged to isolate stromal vascular fraction (SVF). Finally, the SVF is cultured and adherent cells are analyzed for presence of cell surface markers through flow cytometric analysis to confirm the presence of mesenchymal stem cells characteristics.

The first attempt to isolate ASCs is initiated by appropriate washing, followed by their digestion with collagenase and centrifugation to separate stromal vascular fraction (SVF). The SVF is considered as a source of adipocyte progenitors and ASCs along with other cells; iterative sub-culturing enriched the plastic adherent ASCs (Figure 1) [88,89,90].

Additionally, this process has been further modified to recover ASCs from human adipose tissues [91,92,93,94,95]. Centrifugation speed affects the cell yield and 1200× *g* has been observed as optimal centrifugation speed for sufficient recovery of cells [96]. The general procedure of isolation of ASCs initiates from fragmenting large adipose tissues into smaller tissue chips and to avoid connective tissues as they might become source of contamination; this is followed by washing adipose tissues with phosphate-buffered saline (PBS)/Dulbecco’s phosphate buffered saline or saline to remove blood; wash buffers can be supplemented with antibiotic/antimyocotic [97]. The properly rinsed tissue is further minced in sterile condition and then washed again with PBS to remove any traces of blood. The minced tissue is incubated with 0.075–0.5% collagenase type IA at 37 °C for 30 min [68,97]. Another study used collagenase type I (0.5 mg/mL) in equal volume of adipose tissues to digest adipose tissue [98]. Collagenase type II and type IV might also be used; however, optimum concentration of enzyme depends upon quality of enzyme [97]. In addition to collagenase a recent study showed that trypsin can be a cheaper alternative for digesting adipose tissues [99]. Enzymatic activity of collagenase or trypsin is negated by supplementing digested tissue sample with DMEM or α-MEM supplemented with 10% or 20% inactivated fetal bovine serum (FBS) [53,97]. Notwithstanding the enzymatic digestion is a costly method for extraction of ASCs and might affect efficacy and safety [100,101,102]. Therefore, the recent study has explored the economical non-enzymatic method for standardization of ASCs isolation [103]. In another study, lipoaspirate was cultured without enzymatic digestion and sub-cultured after five days; suspension cells were removed from culture flasks by washing and only adherent cells were further analyzed for mesenchymal stem cells characteristics [104]. Similar to this study, another attempt was made to develop non-enzymatic method by simple washing and excessive and repeated shaking of adipose tissues to collect infranatant, which was further centrifuged and collected SVF was cultured to grow ASCs [105]. Notably, this study reported no major differences in cell characteristics isolated from enzymatic and non-enzymatic methods; however, cellular yield was higher in the enzymatically digested method. In another recent study, the mesenchymal stem cells (MSCs) obtained from harvested adipose tissue of animal or human were pluripotent and successfully differentiated into adipocyte and osteoblasts [106]. Various commercial mechanical devices have been developed to process adipose tissue; which uses forces, such as pressure, centrifugal force, shear force, radiation, and ultrasound, etc. to disintegrate the tissues [107]. To maintain sterility, safety, and quality of ASCs and to fulfill the regulatory requirements, various attempts have been made to develop closed and sterile isolation system to reduce uncertainty [107]. However, more extensive studies are required to set standard protocol to fulfill the clinical regulation to explore real-time therapeutic effectiveness of ASCs.

## 3. Characterization of ASCs

Ability of colony formation of stem cells is an indicator of potency and proliferation [108,109]. When stem cells are cultured in low density, each cell have capacity to form individual colonies [110]; however, stem cells that are isolated from rat or mouse may form more than one colony, as the cells may disintegrate from colony and regenerate another cell colony [111,112,113]. CFU can be determined by culturing the cells in medium for 10–14 days, after which thier colonies are visualized and counted using crystal-violet stain. Similarly, cells are also characterized based on expression of their surface markers by using flow cytometry [114]. Characterization of surface markers of ASC is generally carried out by incubating subcultured cells with primary monoclonal and secondary antibodies that are labeled with dyes, such as fluorescein isothiocyanate (FITC), texas red, allophycocyanin (APC), or phycoerythrin (PE) [115,116]. Further, these cells incubated with labeled dye conjugated secondary antibodies are washed. The minimum suggested for positive markers are represented in Table 1.

However, there is a great discrepancy and inconsistency in the data available about expression of CD34 in ASCs. It has been widely accepted that CD34 is not present on the surface of cells; however, this observation is largely based on cultured MSCs, not the tissue-resident MSCs [118]. The evidences have shown that CD34 is present in freshly isolated ASCs and disappear after several passages [30,118]. Notably, the presence of other surface markers, such as HLA-ABC, HLA-DR, SH2, SH3, STRO-1, VEGF2, vWF, ABCG2, SSEA-1 (CD15), PDGFR, α-SMA, c-Kit (CD117), OCT4^+^, and CCR5X (CD195) have also been reported [117]. Further, after corroborating the surface markers, cells are characterized based on their differentiation potential in chemically-induced medium.

### 3.1. Differentiation Potential of Adipose-Derived Stem Cells

**Figure 2:**
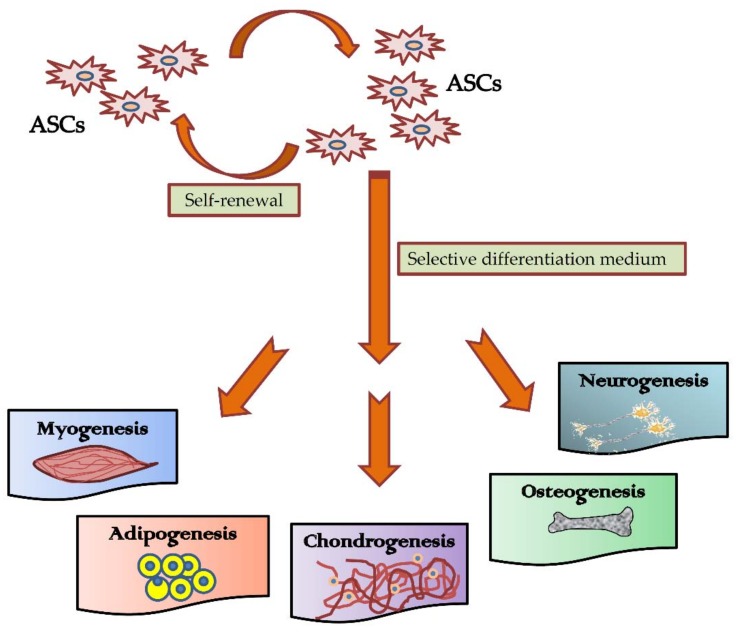
Multi-differentiation potential of ASCs.

Multilineage differentiation potential of ASCs towards both mesenchymal and non-mesenchymal lineage cells have been reported [119]. This may be achieved by the introduction of factors promoting specific lineage (Figure 2) [53].

#### 3.1.1. Osteogenic Differentiation of ASCs

ASCs has potential to differentiate into osteoblasts in presence of limited number of cytokines; which provides opportunity to address bone related disorders within short-time period [120]. Osteogenic medium contains inducing factors, such as dexamethasone, ascorbic acid/ascorbate 2-phosphate, cholecalciferol, and β-glycerophosphates [121,122,123,124] in combination with factor, such as transforming growth factor-β (TGF-β), vitamin D3, and bone morphogenetic proteins (BMPs) [125,126]. Recent studies have described role of quercetin, a natural flavonoid, in up-regulating *Osx*, *Runx2*, *BMP-2*, *Col-1*, *OPN*, and *OCN* genes, promoting the osteogenesis of mouse and human ASCs [127,128]. Osteogenic differentiation is regulated by transcription factors, such as core binding factor-1 alpha (CBF-1α), runt-related transcription factor 2 (Runx2), osterix, homeobox protein Hox-B7 (HOXB7), Hoxa2, Hoxa9, core binding factor-β (Cbf-β), olyma enhancer binding protein 2β (Pebp2β), Sox9, TNF-α, FOXC2, PPARγ, YAP, MyoD, BMP9, β-catenin GATA4, and GATA6 [129]. Moreover, two factors HIF-1α and TWIST have been reported for their inhibitory effect on osteogenic differentiation through their interaction with Runx2. Transforming growth factor-β (TGF-β)/bone morphogenetic proteins (BMPs), Wnt/β-Catenin, Notch, Hedgehog, and fibroblast growth Factor (FGF), etc. are reported as major signaling pathways in regulating osteogenic potential of ASCs [121]. Dexamethasone activates FHL2/β-catenin-pathway to induce over-expression of RunX2 and collagen type I alpha 1 (COL1A1); whereas, ascorbic acid promotes the secretion of collagen type I to increase the activation of integrin-mediated signaling and β-glycerophosphate provide phosphate resources to up-regulate the expression of osteogenic gene [130]. Vascular endothelial growth factor A (VEGF-A) plays a crucial role in bone regeneration due to its potential to promote both angiogenesis and osteogenesis in human ASCs [131]. A combined treatment of ASCs with VEGF and BMP-2, -4, -6, and -9 have demonstrated to promote osteogenesis through over-expressing osteogenic alkaline phosphatase gene [132,133]. Moreover, a recent in vitro study reported that BMP2 exert no significant and constant effect in the promotion of osteogenesis [134]. Similarly, no catalyzing effect of BMP2 have been reported on osteogenesis of hASC in presence of ascorbic acid and β-glycerophosphate [135]. On contrary, BMP2 has been reported for its synergistic effect on vitamin D3 in the promotion of osteogenesis of ASCs [136]. Interestingly, hypoxia in addition to promoting angiogenesis [137], has also been reported to enhance osteogenic potential and up-regulate the expression of octamer-binding transcription factor 4 (OCT4), Kruppel-like factor 4 (KLF4) and NANOG [138,139,140]. However, the inhibitory activity of hypoxia against mineralization and osteogenic potential of ASCs via IGFBP3 up-regulation have also been documented [141]. The hypoxia also inhibit the alkaline phosphatase activity, expression of core binding factor α-1 (CBFA-1), and osteopontin leading to negative regulation of osteogenic potential of ASC [142]. During osteogenic differentiation, the mitochondria get activated to fulfill high energy demands in necessary biochemical reactions [143]. Sirtuin, such as Sirt1 and Sirt 6, also plays a crucial role in osteogenic and chondrogenic potential of MSCs [144,145]. Additionally, bone morphogenetic protein (BMP), a cytokine inducer is promptly used to direct osteogenic differentiation among ASCs [146], and the significance of BMP-2 and BMP-7 have been clinically accepted in Australia, United States, and Europe [147]. BMP-2, BMP-6, and BMP-14 are considered as major factors in osteogenic differentiation of ASCs s [148,149]; whereas, BMP-7 promotes both chondrogenesis as well as osteogenesis [150]. The osteogenic potential of ASCs is affected by the concentration of BMP and nature of differentiation medium [151,152,153]. A notable osteogenesis promoting effect of combined retinoic acid and BMP2 in murine ASCs have also been evidenced [154].

It has also been postulated that BMP alone is insufficient to direct MSCs to differentiate into osteogenic lineage; as it triggers both adipogenesis and osteogensis at an equal rate [146]. Specifically, BMP signaling pathway activates with binding of ligand to heterodimeric serine/threonine kinase BMP receptor, which triggers the activation of Smad-dependent signaling pathway (Smad1/5/8) and Smad-independent signaling pathway (JNK, p38); mediating both adipo- and osteogenesis [146]. However, the heterodimer of Smad4 with phosphorylated transcription factors Smad1, Smad5, or Smad8 activates the expression of osteogenic promoting genes of ASCs [121]. BMP also regulates expression level of other osteogenic factors, such as core-binding factor-1/Runt-related family 2 (Cbfa1/Runx2) [155]. Besides, the Wnt5a directs osteogenic differentiation through Wnt signaling pathway and suppress PPAR-γ in ASCs [156]. This pathway activates β-Catenin–T-cell factor/lymphoid enhancer factor (TCF)/Lef transcription factors (Lef) which further enhances osteogenesis [121]. A similar behavior of endogenous cytokine, such as tumor necrosis factor-alpha (TNF-α) has also been observed [157], where it mediates its effect through activation of nuclear factor-κB (NF-κB) and inhibit PPAR-γ function; TNF-α also promotes expression of TAZ (transcriptional coactivator with PDZ-binding motif) leading to osteogenic differentiation of ASCs. Beside BMP and Wnt signaling pathway, the notch signaling route has also been reported for its role in osteogenic differentiation of stem cells through sequential release, nuclear transportation, and assembly of Notch intracellular domain (NICD) into nuclear transcription factor, leading to cascade of events regulating the expression of osteogenic genes [158]. Apart from these pathways, accumulation of reactive oxygen species (ROS), an indicator of oxidative stress, have also been reported to suppress the osteogenic potential of MSCs.

#### 3.1.2. Chondrogenic Differentiation of ASCs

Adipose-derived stem cells (ASCs) have been shown to exhibit similar chondrogenic potential as bone-marrow derived stem cells [28,159]. However, recent studies have suggested that inclusion of cytokine, such as BMP-6 and a higher concentration of other growth factors in culture medium, improvise the chondrogenic potential of ASCs [160,161]. Furthermore, the presence of ascorbic acid phosphate, dexamethasone, bovine serum albumin, linoleic acid, sodium pyruvate, transferrin, selenous acid, proline, l-glutamine, and TGF-β1 have also been reported for their chondrogenic promoting activity in vitro [126,129,162,163]. In addition to this, the transcription factors, such as SRY-related high mobility group-box gene 9 (*Sox9*), Zinc-finger protein 145 (ZNF145), HOXD9/10/11/13, FOXO3 A, Wnt 11, and STAT3 play an active role in chondrogenesis [129]. However, some other transcription factors such as HOX2a, Smad3 and YAP down regulate the chondrogenic differentiation potential of MSCs. Scaffold- and pellet-based culture systems provide three-dimensional (3D) support, high culture density, and microenvironment for chondrocytes differentiation, leading to cartilage generation [164,165]. Micropellets are used as high-density culture system (2.5 × 10^6^ cells/pellet) to promote cellular interaction for the development of cartilage like structure [164].

The ASCs tend to grow as a monolayer in in vitro and avoid cell-cell contact by growth inhibition. However, excessive cell accumulation, as occurring in high-density micropellets, is a fundamental prerequisite for chondrogenic differentiation. In recent years, three-dimensional (3D) constructs, such as scaffolds, various hydrogels, alginate gels, and matrices, have been developed to mimic the physiological milieu and overcome growth inhibition [12]. Similarly, scaffolds that are covered with different chemotactic agents, as well as matrices of varying stiffness values, have been designed to achieve the directional migration of cell cultures. In 2007, Xu et al. were among the first groups to focus on mechanical properties of chondrocyte differentiation in a 3D mass model [46]. Hydrogels of polymers, such as agar, alginate, and agarose are also used to provide structural support, mechanical stimuli and micro-environment to direct chondrogenesis [166,167,168]; however, continuous interaction of cells with hydrogels may cause cellular sensation [169]. The Dulbecco’s modified Eagle’s medium (DMEM) is generally used as basal medium that is used in cell culture, which is supplemented with ascorbate-2-phosphate, insulin, TGF-β1 and 1% FCS [28]. However, even in absence of FCS, the DMEM when supplemented with TGF-β3, insulin, transferin, albumin, dexamethasone, and ascorbic acid promoted chondrogenic differentiation of ASCs [170]. Besides, the effect of oxygen concentration seems confounding; still, its concentration needs to be properly regulated to direct chondrogenesis in ASCs [166]. Molecular techniques, such as real-time PCR, western blot analysis, ELISA, and RNA microarray are used to study the expression of chondrogenic genes, such as *collagen I/II/VI/IX/X*, *COMP*, *HAPLN1*, *SOX 9*, *matrilin 3*, *Indian hedgehog*, *homeobox 7*, *chondroadherin*, *WNT 11*, *aggrecan*, *alkaline phosphatase*, *fibromodulin*, *osteocalcin*, and *PTHrP* during osteogenesis of ASCs [166,167]. Additionally, staining of ECM with alcian blue, toluidine blue, or safranin-o are simple methods to determine the chondrogenic potential of ASCs.

#### 3.1.3. Adipogenic Differentiation of ASCs

Adipogenic potential is considered as an exclusive characteristic to determine the quality of ASCs. The adipogenesis is directed by using differential medium enriched with isobutylmethylxanthine (IBMX), indomethacin, 3-Isobutyl-1-methylxanthine (IDII), insulin, and dexamethasone at varying concentrations [53,171,172]. During adipogenic differentiation, MSCs are firstly directed to differentiate into preadipocytes and then to adipocytes [173]. The effect of dexamethasone on adipogenic differentiation depends upon factors, such as time and concentration [171]. The prolonged exposure of dexamethasone promotes adipogenesis and curtails osteogenesis in MSCs [174]. At high concentration, insulin behaves like insulin growth factor 1 (IGF-1) and it promotes differentiation and the proliferation of preadipocytes [175]. Hydrocortisone is another glucocorticoid agonist along with dexamethasone which initiate the signal cascade to activate preadipocyte receptors and their differentiation into adipocytes in the presence of insulin [176]. IBMX along with dexamethasone activates protein kinase A (PKA) signaling pathway directing the transcription of PPARγ, and finally leading to adipogenic differentiation [177,178]. The ASCs differentiation is primarily regulated through receptor tyrosine kinases (RTKs) by Akt and extracellular ERK-1) signaling pathways; in which Akt activity promotes adipogenesis; whereas, ERK-1 negatively regulates adipogenesis [179]. It has been also reported that high cell density and structural support also promotes adipogenic differentiation through paracrine and autocrine actions [180,181]. Similarly, obestatin mediates its adipogenic differentiation via autocrine and paracrine activities [177]. PPARγ agonist, such as rosiglitazone, troglitazone, pioglitazone thiazolidinediones, or glitazones might also be useful to enhance the adipogenesis in vitro [53,171]. In addition to transcription factors, such as PPARγ1, PPARγ2, and EBF-1; other factors, such as PRDM16, Twist-1, Dermo-1, COUP-II, Sox2, and Oct4 promote adipogenic differentiation; whereas, GATA2, Foxa1, and HOXC8 downregulate the adipogenesis of MSCs [129]. Furthermore, C/EBP-α, C/EBP-β, and C/EBP-δ regulate the transcription of PPARγ to modulate adipogenic differentiation of ASCs [182]. Cell culture models have indicated that BMP4, Wnt signaling, cell shape, and density also induce adipogenesis in MSCs [183,184]. On the other hand, though previous studies have reported potential of BMP2 and BMP-7 to form fat cells, the role of BMP in induction of adipogenesis is not well understood, and thus, it is not considered as an integral component of adipogenic differentiation medium [171]. After the cells grown in adipogenic differentiation medium, they are fixed in 10% formalin solution or 70% ethanol to determine their lipid content by staining with dyes, such as Oil Red-O, neutral lipid fluorescent dye, or nuclear fluorescent dye at room temperature [53].

## 4. ASC Secretome and Its Therapeutic Effect

**Figure 3:**
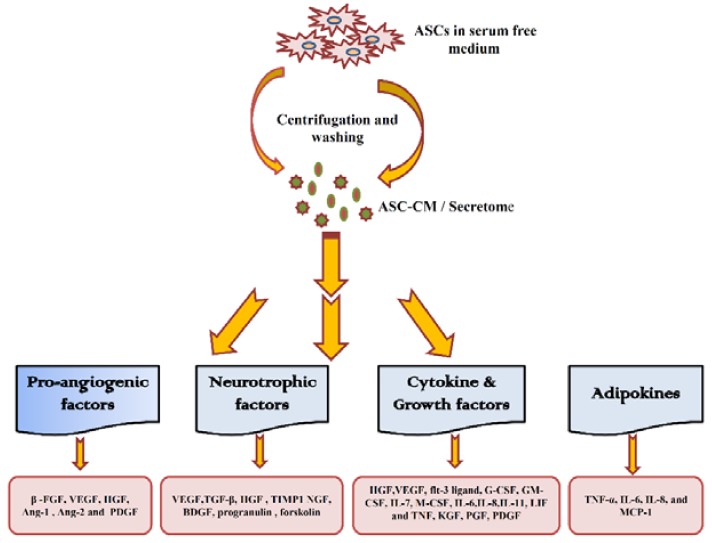
ASC-secretome. The Secretome is highly rich in cytokines, growth factors, angiogenic factors, adipokines and neurotrophic factors, which enables ASCs to regenerate and repair injured/diseased tissues.

ASCs regeneration potential and therapeutic values also lies in its secretome, which is rich in extracellular proteins and growth factors (Figure 3) [185].

This secretome exert varying beneficial effect through the paracrine activity of ASCs [186]. The pro-angiogenic factors in secretome mainly includes PDGF, FGF, VEGF, HGF, angiopoietin (Ang-1 and Ang-2), of which PDGF are present in higher concentration [187,188]. Cell secretome is harvested from the cells that were cultured in serum-free medium for 12 h to 48 h [189]; and their level is determined using techniques, such as two-dimensional (2D) and difference gel electrophoresis, mass spectrometry and ELISA [190]. Other techniques, like stable isotope labeling by amino acid in cell culture (SILAC), isobaric tags for relative and absolute quantitation labeling (iTRAQ), western blot, 2D planar arrays or 3D bead systems have also been employed [191]. Proteins of secretomes are mainly associated with cytoplasm, nucleus, endoplasmic reticulum, and ECM [185]. These proteins assist and regulate cellular metabolic activity, cell signaling, DNA repair, cytoskeletal development, and mitosis. In the mouse model, the secretome of human ASCs conditioned medium (hASCs-CM) restored cytokine balance and reduced the diabetic pain [192]. Another study reported that ASCs-CM enhances the collagen synthesis and migration of dermal fibroblast to improve wrinkling and wound healing in the animal model [193]. The hypoxic condition has also been known to influence the characteristics of stem cells, including their secretome and efficacy. In the interesting reports, hypoxia increased the rate of proliferation of ASCs and accelerated their wound-healing function through the up-regulation of VEGF and bFGF [194,195]. Wang et al. documented that hypoxic condition (5%) increased the differentiation of ASCs toward the smooth muscle phenotype [196]. Hypoxia also augmented the migration potential of ASC by enhancing the expression of stromal cell-derived factor (SDF)-1 [197]. Besides, the other secreted growth factors, like keratinocyte growth factor (KGF), TGF-β1, HGF, and VEGF of conditioned medium also might play a crucial role in wound healing. Ribeiro et al. revealed an increased neuronal cell density and its metabolic activity by introducing ASCs secretome supplemented with growth factor bFGF and B27 [198]. A recent study has reported that sphingosine-1-phosphate (S1P) and cytokine of ASCs secretome control the inflammation of central nervous system [199]. According to Constantin et al. ASCs secretome containing bFGF, PDGF-AB, and brain-derived growth factor controlled the experimental autoimmune encephalomyelitis (EAE) [200]. Reports have also evidenced that secretion of VEGF, TGF-β, and hepatocyte growth factor (HGF) promote angiogenic and neurogenic responses [185,201]. Further, the released tissue inhibitor of metalloproteinase-1 (TIMP-1) and progranulin provide neuroprotection potential to ASCs [202]. In this concord, IGF-1 and BDNF have been shown to improve the functional recovery in learning and behavior in rat model [203]. The in vitro study also indicate that ASCs plays a crucial role in tissue regeneration through NGF-induced activation of 5′ AMP-activated protein kinase (AMPK) [204]. A recent study has demonstrated that the BDNF upregulated the axonal growth in CNS [205]. Besides, the ASC-CM mitigated the oxidative stress in stressed SH-SY5Y neuron-like cells and restored cell morphology, viability, and electrophysiological activity [206]. This restructuring activity was linked with the presence of antioxidant and growth factors, like BDNF, glial cell line-derived neurotrophic factor, and TGF-β1. Another study indicated that VEGF-A and VEGF165b derived from ASCs and ADSC-CM were effective in reducing the pain level in oxaliplatin-treated neuropathic rats [207]. It has been reported that mechanical stress enhance the secretion of VEGF, G-CSF, HGF, Leptin, IL-8, PDGF-BB, Angiopoietin-2, human umbilical vein endothelial cell (HUVEC) migration-stimulating factors, and follistatin [208]. Further, the oxidative stress and hypoxia also increased the level of VEGF, IL-8, leptin, angiopoietin-2, and PDGF-BB in cell culture medium.

The cytokines in human ASCs secretome mainly includes, angiogenic, hematopoietic, and proinflammatory cytokines, like HGF, VEGF, flt-3 ligand, G-CSF, GM-CSF, IL-7, M-CSF, IL-6, IL-8, IL-11, LIF, and TNFα [209]. ASCs also secrete adipokines such as FGF, ILs, IGF-binding protein, PDGF, TGF-β, TNF-α, and VEGF [210]. However, the adipokines like TNF-α, IL-6, IL-8, and MCP-1 have been reported to promote tumor growth [211]. The role of ASCs in regulating breast cancer is confounding due to varied nature of secreted adipokines, such as CCL5, which enhances the motility of MCF-7 breast cancer cell in vitro [212]. In contrast to this, another study reported that high density ADSC-CM inhibited the MCF-7 [213]. Though the contradictory impact of ASCs and its secretome is wide in literature, it has been proposed that ASCs might only promotes cancer in active breast cancer cells [214]. Wang et al. reported that ASC-CM significantly improved cellular proliferation, regulated apoptosis and cellular senescence in UVB irradiated human dermal fibroblasts (HDFs); which indicates the protective role of secretome against damages that are caused due to aging [215]. Similarly, TGF-β1-treated ASCs-CM upregulated type I collagen and promoted proliferation and mobility of skin fibroblasts in mice model indicating the role ASCs-CM in wound healing [216]. The ASCs-CM human antimicrobial peptide LL-37 treatment also improved the migration of HDF [217]. The presence of VEGF, bFGF, TGF-β1, TGF-β2, HGF, keratinocyte growth factor (KGF), PDGF-AA, placenta growth factor (PGF), type I collagen, fibronectin, and superoxide dismutase (SOD) in ASCs seretome was effective in improving skin texture and wrinkle in micro pig model [218]. In addition, another study evaluated the potential of secretome (concentrated ASCs-CM) in controlling ischemia reperfusion (IR) injury in mice model indicating the potential ASC-secretome in providing therapeutic option for treatment of IR injury [219]. The ASCs–CM has also recovered gastric wound in rat model through promoting angiogenesis and re-epithelization [220].

Along with growth factors and cytokines, ASCs also secrete exosomes of 30–150 nm size [221]; which mediate the signaling effects and mimic the functional characteristic of cells [222,223]. Studies revealed that the exosomes of ASCs (over-expressing Nrf2) have improved wound healing in diabetic foot ulcer rat model [224]. In a seminal report, the exosomes derived from ASC isolated from cancer patient have been partly attributed for their therapeutic effect, indicating that expanded ASC remain unaffected by patient condition [225]. Choi et al. demonstrated that the ASCs-exosomes enriched in micro-RNA improvised the regeneration of human dermal fibroblasts [221]. Similarly, ASCs-exosomes when engulfed by fibroblasts promoted soft tissue repair and cutaneous wound healing [226]. Furthermore, the intravenously administered ASCs-exosome regulated the ratios of collagen type III: type I, TGF-β3:TGF-β1 MMP3:TIMP1, fibroblast differentiation, and thereby reduced scar size in the murine model [227]. In a report by Lee et al., ASCs-secreted exosomes demonstrated therapeutic potential against Huntington’s disease by considerably reducing the aggregation of mutant Huntingtin protein, mitochondrial dysfunction, and cellular apoptosis in R6/2 mice-derived neuronal cells [228]. These exosomes have also improved the efficacy of anti-cancer drug in mouse model of hepatocellular carcinoma and promoted the migration of breast cancer cell line (MCF7) [229]. Similarly, various studies have documented the therapeutic activities of ASC released exosomes against neurodegenerative and vascular diseases [230].

Based on above body of evidence, the ASCs-derived secretome seems to be a potential agent for the treatment of various disorders.

## 5. Conclusions

Adipose tissues are considered as most promising and enriched source of ASCs, and the easy harvesting procedure and less ethical complexities, makes ASCs the most appropriate stem cell source in development of regenerative therapeutic approaches. These cells exert their beneficial effect not only through differentiation, but also through the paracrine effect of secretome. However, the extensive studies are needed to understand the nature of secretome of ASC and their specific role in regeneration and repair of damaged/diseased tissues.

## Figures and Tables

**Table 1:** List of minimum mesenchymal stem cells (MSC) immunophenotypic markers on ASC [117].

ASC Immunophenotypic Surface Markers	
Positive (+ve)	CD90, CD44, CD29, CD105, CD13, CD73, CD166, CD10, CD49e and CD59
Negative (−ve)	CD31, CD34, CD45, CD14, CD11b, CD19, CD56 and CD146
